# AI powered ELT: Instructors’ transformative roles and opportunities

**DOI:** 10.1371/journal.pone.0324910

**Published:** 2025-05-29

**Authors:** Afnan Almegren, Rehan Megren Almegren, Abduljalil Nasr Hazaea, Hassan Saleh Mahdi, Jamal Kaid Mohammed Ali

**Affiliations:** 1 Department of Applied Linguistics, College of Languages, Princess Nourah bint Abdulrahman University, Riyadh, Saudi Arabia; 2 Department of English Language Skills (PY), Najran University, Najran, Saudi Arabia; 3 Faculty of Language Studies, Arab Open University, Saudi Arabia; 4 Department of English Language and Literature, College of Arts and Letters, University of Bisha, Bisha, Saudi Arabia; Ahvaz Jundishapur University: Ahvaz Jondishapour University of Medical Sciences, IRAN, ISLAMIC REPUBLIC OF

## Abstract

The integration of artificial intelligence (AI) in education is reshaping English language teaching (ELT), redefining the ‘assistant’ function of technology and the ‘interaction’ roles of language instructors. This study specifically investigates instructors’ roles in AI-powered learning environment and its benefits for ELT. It develops a conceptual framework for AI powered ELT. Using a qualitative approach, data were gathered through online focus group discussions with 22 instructors from different regions. The thematic analysis revealed that AI empowers ELT by enabling personalized teaching, mono-active language practice, increased productivity, automated feedback and assessment, and adaptive material creation. Additionally, instructors assume four key roles: evolving facilitators, blended role practitioners, flexible material designers, and creative prompters. These transformations reflect a departure from the assistant function and the facilitator roles cantered on human interaction to more mono-active roles aligned with AI-powered innovations. The study highlights the necessity for ELT to embrace these new roles to effectively navigate the evolving dynamics of ELT in the different region in the AI era. Acknowledging its qualitative limited scope, the study recommends further broader research on these transformations with specific attention to language skills.

## Introduction

The relationship between AI technology and language education is synergistic. This relationship presents a transformational shift in the way teachers teach a language [1]. AI technologies are increasingly integrated into educational settings, transforming traditional teaching and learning paradigms. Therefore, AI-based teaching environment requires teachers to cope with what is required from them in this teaching mode [2,3,4]. AI based teaching has the potential of supporting language education through having personalized tools that are efficient and help both learners and teachers [5]. AI-powered tools such as adaptive learning platforms, chatbots, and automated assessment systems are becoming prevalent, supporting real-time feedback, and enhanced student engagement [6].

In the context of higher education, AI is perceived as a double-edges sword. Some educationalists look at the substantial benefits that AI apps play in the learning and teaching processes [5,3,7]. Others focus on the devastating impact of AI tools on the students’ creativity and authenticity [e.g., 8]. For those who support the integrity of AI in education, they highlight the many potential benefits supposing that AI offers immediate feedback, personalized learning and develop students’ competence in the learning process [7].

Similarly, in ELT, AI provides benefits for students including improvements in speaking, writing, reading, pedagogy, and self-regulation [9]. AI participates in enhancing students’ phonology, syntax, and comprehension skills [10]. AI technologies can significantly change ELT by providing intelligent tutoring systems, personalized training, and automated grading. However, ensuring successful integration of AI in education relies largely on the instructors’ expertise, adaptability and direction [4]. On the other hand, the other team maximizes the potential harness of AI on students and teachers over its substantial benefits. For example, AI is responsible for job displacement [8,11]; AI kills creativity among learners [12]. Despite the potential of AI in revolutionizing English language learning, the role of instructors remains indispensable [13]. Understanding instructors’ perspectives on AI-based learning is crucial for maximizing the effectiveness of these technologies and addressing the challenges they present.

The potential benefits and challenges of AI have been widely studied over the past few years [14,15] among many. The research interests then have been transformed to focus on the ethical considerations that must not be neglected while integrating AI in the learning process [16,17]. Currently, scholarly calls for analysing the future of integrating AI in ELT technology specifically how to bring fun to the learning situation [17]. In responding to this call, we in this study probe at the potential opportunities of integrating AI in the ELT, and the impact of AI in reshaping the role of language instructors in the teaching process.

Thus, this study seeks to investigate the role of language instructors and the potential of AI technology in ELT in an AI powered English language teaching (AIPELT). It specifically aims to:

1. identify the transformational roles of English language instructors in AI-powered learning environment; and
2. explore the opportunities of AI-powered technology in enhancing ELT at universities.

## Literature review

### Instructors’ roles with TALT

Technology-based paradigms reshaped the roles of language instructors. When discussing the roles of language instructors with technology assisted language teaching (TALT), we are to differentiate between four broad roles: teacher-as source of knowledge, teacher as facilitator of knowledge, teacher with multi-literacies, and the potential role with AI.

In traditional learning, teachers are the knowledge suppliers to their students. However, this role seemed to contradict with students’ preference. Theories like social constructivist called teachers to be mediators rather than disseminators of knowledge [18]. On the contrary, in line with computer-assisted language learning (CALL), students’ centred learning-another learning situation stemmed from communicative approach. Teachers played various roles as facilitators, guider, co-learners and the like [19]. Márquez [20] argued that EFL instructors evolve role as advisors, social workers and supporters. Furthermore, with mobile-assisted language learning (MALL), language instructors’ pivotal role in English education is indispensable [21]. This shift emerged with online learning. The online learning modes of teachers are required to play various roles in addition to the previous ones. Hung and Lee [22] recommended that instructors to adopt social, pedagogical, technical and managerial roles. This, getting professional knowledge in using technologies is a must. With the emerge of AI technologies, the role of instructors may change and play other roles different from the revised role in the three previous modes. This radical shift questions the interaction role of instructors in ELT. It also scrutinizes the assistance role of technology in ELT.

### Instructors’ roles in AI-powered ELT

Research indicates that while AI can automate routine tasks, instructors play a critical role in interpreting AI data, providing emotional support, and fostering higher-order thinking skills [23]. The synergy between AI tools and human instructors can enhance learning outcomes.

The instructors’ transforming roles in AI-enhanced learning have been the interest of much current research [2,3,4,24]. Instructors create AI-based content; they assist students to steer these tools [4]. Instructors in AI-generated virtual learning enhance fostering and motivating positive emotions among students [3]. In online setting, the roles of instructors remained pivotal [2]; they can promote students’ life skills by playing roles as designers [24]. Ramakrishnan et al. [4] reviewed the role of instructors in AI-based education. The study reported that teachers help students navigate AI tools and create relevant learning materials. Teachers guide students while traverse AI-powered platforms. They also direct students develop the learning outcome by levering AI-powered sources. Teachers also curates learning AI-based content. Gómez-Rey et al. [24] examined the important role of instructors in the 21^st^ century. They used interview, literature review, pilot study to collect data form 925 students. The study reported a new role where instructors are suggested to be life skill promoter. They study also revealed that instructors in online learning should be designers for online programs and task. They are pedagogues, managerial and technical. Furthermore, Golub et al. [25] confirmed that human instructors are irreplaceable in AI-based education. They organize the learning process. For them, successful teaching depends on the alignment between conventional and AI powered technology which is guided by experienced teachers.

Previous studies have not clearly sopped the role of instructors in AI based learning. On the contrary, some studies focused on the roles of AI in the learning process [24], Ramakrishnan et al. [4]. All previous efforts were about pinpointing the advantages and disadvantages of this AI tools. It seems that interaction approaches to ELT are shifting towards a mono-action approach to AI-powered ELT. Therefore, in this study we try to highlight the transformative roles of ELT featured by AI-technology.

### Opportunities of AI in ELT

Integrating AI in English Language Learning (ELL) at universities played protentional and transformational role as some studies reported [8,26,27]. AI applications in language learning include adaptive learning systems, natural language processing (NLP) tools, and intelligent tutoring systems [28]. AI provides automatic feedback, supports individual learning and gives space for language practising [27]. AI tools offer language learning apps and tutoring systems serve students needs’ and engage them in game-based environment [1]. Adopting AI in English as a second language (ESL) develops students’ grammar, phonology and comprehension skills [10].

AI technologies offer opportunities for practice in immersive environments. Numerous studies took AI as their focus in different situations and for various purposes. Amin [16] examined the Multifaceted impact of AI on EFL learning. The study reviewed some of the potentialities of integrating AI technology emphasising the role of personalized learning, contentious feedback, and real time practise among many. The study emphasized the future of AI in EFL education to be based on the collaboration between instructors and AI technology.

Du and Gao [29] identified and assessed factors influencing the adoption of AI tools using technology acceptance theories. A multi-criteria decision-making model based on the TAM framework, encompassing four main factors and ten sub-factors from previous studies, was proposed. Data from 17 experts were analyzed using the Analytic Hierarchy Process (AHP) to prioritize these factors. The study found that effectiveness, efficiency, and complexity were the most influential factors encouraging AI adoption, while perceived fees and rewards were less significant. Perceived time, flexibility, and pleasure were of intermediate importance. The article also provides insights into teachers’ experiences with AI application adoption processes.

Mohamed [12] investigated the perceptions of ten EFL faculty members at a Saudi university regarding the effectiveness of ChatGPT in supporting English language learning. Through in-depth interviews, the study found that faculty opinions varied. Some saw ChatGPT as useful for providing rapid and accurate responses, while others worried it might hinder students’ critical thinking and research skills and could reinforce biases. Despite these concerns, the faculty recognized ChatGPT’s potential to complement traditional teaching methods and recommended further experimental research to evaluate its effectiveness. Overall, the study highlights ChatGPT’s potential in enhancing EFL students’ English proficiency.

Crompton et al. [9] explored the challenges and benefits of using AI in ELT. Recognizing the importance of English for various global interactions, the research conducted a systematic review following PRISMA principles, identifying 42 relevant studies. The findings detail the geographical locations of these studies, the ages of learners, and the years of study. Through grounded coding, the study identified AI’s benefits in ELT, including improvements in speaking, writing, reading, pedagogy, and self-regulation. However, challenges such as technology breakdowns, limited AI capabilities, fear of AI, and concerns about standardizing language were also highlighted. The study provides valuable insights for policymakers, funders, practitioners, and educational leaders to understand current trends and offers practical implications for future AI use in ELT.

In their systematic review of published research for six years, Sharadgah and Sa’di [30] explored the use of AI in ELT. The study reported that AI in ELT is promising, enhancing language skills, translation, assessment, and learner satisfaction, with most research focused on higher education and students. Identified AI techniques include machine learning, neural networks, and natural language processing, among others.

Karataş et al. [5] explored the use of ChatGPT, an AI application, in foreign language education to fill a research gap by providing insights into its potential, benefits, and drawbacks. Conducted with 13 preparatory class students at a Turkish university’s School of Foreign Languages over four weeks, the study involved integrating ChatGPT into their learning experiences. Thematic analysis of interview data revealed that ChatGPT positively impacted students’ learning, particularly in writing, grammar, and vocabulary acquisition. It also enhanced motivation and engagement through its versatile and accessible nature in various learning activities. These findings offer valuable insights for educators and researchers in developing effective teaching strategies and curricula incorporating ChatGPT technology.

Despite these advancements, AI in ELT is still nascent, with gaps such as insufficient attention to non-verbal cues and vague AI definitions. The review recommends practitioners familiarize themselves with current AI tools to design effective classroom systems. Researchers are urged to provide detailed methodologies and address existing challenges to optimize AI’s role in ELT.

## Methods

### Research design

A qualitative approach is employed to gather deep, rich and comprehensive data on the role of language instructors with the advent of AI from instructors’ perspectives. This approach facilitated the collection of detailed and nuanced data to understand instructors’ roles on integrating AI and its potential benefits for ELT.

### Instrument

Data was collected through focused group discussions conducted online via ResearchGate. As its name indicate, this platform is considered a gate for researchers from around the world to share their research publications. It also enables researchers to conduct open discussions through posting questions and answers for timely issues. The platform is an authentic source for such discussions [31,32]. Identities of researchers are evident in their usernames, publications and their contact information such as emails. The focused group discussion on ResearchGate enabled random recruitment of participants, ensuring diversity in viewpoints. The discussions were designed to elicit in-depth insights into the potential opportunities and the instructors’ roles within AI-driven educational paradigms (Appendix 1)

### Participants

Twenty-two instructors from diverse linguistic and academic backgrounds participated in the study. They were conveniently responded to the query on ResearchGate. The participants represented a range of academic ranks, i.e., instructors, PhD candidate, assistant professors, associate professors and full professors. They belonged to various disciplines including Applied Linguistics, ELT, and Education. They came from different regions where they provided a broad spectrum of insights including Middle east and north Africa (MENA), Europe, Australia, and South Asian countries. They come from various cultural multilingualism. Five participants came from Europe, including Serbia, Norway, Italy, and Turkey and the others affiliated to universities in MENA including Saudi Arabia, Yemen, Iraq, Iran, Oman, and Turkey and Libya. Two participants were form South Asian countries including Indonesia and India and one came from Australia (Appendix 2).

### Procedures for data analysis

Thematic analysis was employed to analyze the data systematically. The process involved the following steps as provided by Braun and Clarke [33]. First, key statements and ideas were identified and coded based on recurring themes. Next, related codes were grouped to form overarching themes that captured the core ideas expressed by participants. After that, sub-themes were reviewed and refined to ensure accuracy and relevance to the research objectives. Finally, the themes were interpreted in the context of the existing studies and the broader implications for AI integration in education. This systematic approach ensured that the findings accurately reflect the participants’ perspectives and provide actionable insights for educational stakeholders.

### Ethical and practical oversights

The data were collected from ResearchGate, a publicly accessible platform where participants voluntarily share their insights. No private or sensitive information was obtained beyond what users willingly provided. The research did not involve the collection of personally identifiable information. Any data used were either anonymized or publicly accessible, ensuring that participant confidentiality was maintained. ResearchGate users engage in discussions and share content under the platform’s terms of service, which allows for academic use of shared data as long as it adheres to ethical research principles. The research aligns with standard ethical guidelines that exempt studies from formal ethics approval when they rely on publicly available data and do not involve human subjects in a way that requires informed consent.

## Findings and discussion

This study explored the potential opportunities for AI in enhancing English language teaching at university, and the transformational roles of EFL instructors in AI-powered environment. The findings are presented in line with the research objectives (Table 1).

**Table 1:** Manifested themes on EFL instructors’ role and potential opportunities in AI-powered environment.

Research Objectives	Manifestations
The transformational roles of EFL instructors in AI-powered learning environment	Evolving instructors’ role
	Blended roles
	Material designer
	Creative prompter
The potential opportunities of AI-powered technology in enhancing ELT	Personalized learning
	Interactive language practice
	Increase productivity
	Automatic feedback and assessment
	Materials creation

### Transformational roles of EFL instructors in AI-powered learning environment

The analysis revealed four roles that AI instructors in AI-powered environment. These roles are evolving role, blended role, material designer, and creative prompter. This demonstrates that necessity of EFL instructors to shift from existing roles which are characterized by dominations into having more flexible roles which accommodate with the huge shift in the learning process in AI era.

EFL instructors still have an evolving role in AI-powered environment. That is, they use AI to support their roles. For instance, RN8 confirms, “Our role as empathetic and inspiring educators remains central”. Despite of this huge development of AI in education, teachers’ roles are still primary. RN12 affirms, “AI will not replace the teachers but help them modify and adjust their teaching methods, techniques, and strategies”. RN16 states, “AI will never be able to replace human teachers and instructors in the classrooms”. Furthermore, RN5 adds, “The role of instructors is evolving rather than diminishing”.

EFL instructors to some respondents adopt blended roles in AI-powered environment. To begin with RN17 who initiates, “They foster communication, cultural understanding, and nuanced language skills that AI alone cannot fully replicate”. RN16 mentions, “They guide students through complex linguistic concepts”. RN17 points out, “The teacher will be the one who encourages discussions and promotes critical thinking”. RN18 opines, “They guide each student on an individual learning path that suits their needs RN16 asserts, “instructors can focus on fostering critical thinking, creativity, and deep engagement in the language. Finally, RN12 clarifies, “EFL instructors will remain indispensable in fostering supportive and effective learning”.

It is widely expected from EFL instructors in AI-powered settings to design learning materials to their students. To give an example, RN2 supposes, “EFL instructors are responsible for designing materials, creating content, building life skills, and explaining the ethical and unethical use of such AI applications to their students. RN16 shows, “They design interactive, immersive, and student-centered activities”. Still RN17 claims, “instructors maintain a balance between technological efficiency and human connection in education”.

EFL teachers are also expected to facilitate the learning process to their students. This theme was supported two responses. RN16 says, “They [teachers] are becoming facilitators who use AI to enhance the learning experience”. This is also the perception of RN17 who reports, “The role of the teacher could be a facilitator”.

These various roles that respondents showed demonstrate their understandability of the necessity for EFL instructors to develop their roles as the AI-technology develops. These findings align with Hung and Lee’s [22] recommendation who pointed out that instructors to adopt social, pedagogical, technical and managerial roles. Even though, during the emerged of the communicative approach and the tendency towards learners centred [19], the teachers’ role witnessed modifications. Likewise, our findings are supported by Márquez [20] who argued that in the online learning modes, EFL teachers are required to play various roles like advisor, social worker and supporter in addition to the previous ones.

Among the weird perceptions are the AI may substitute the instructors, but this is not the case at the present time nor will be in the future. However, traditional teachers may not be preferable in this era. This agrees with Golub et al.’s [25] conclusion that human instructors are irreplaceable in AI-based education and that they organize the educational process. For them successful teaching mixes between conventional and AI powered technology.

### The Potential opportunities of AI-powered technology in enhancing ELT

As Table 1 shows, AI-powered EFL teaching provides personalized learning, interactive language practice, increase productivity, automated feedback and assessment, and materials creation. These categories are supported by initial responses.

AI is perceived to provide personalized learning to EFL students. RN1 states, “AI technologies in improving language skills and providing personalized instruction,”. RN8 opines, “AI provides Personalized learning, language learning apps, language learning apps, AI technology offers personalized learning, provides engaging content”. RN7 adds, “These include personalized learning experiences suitable to individual student needs”. RN8 suggests, “It can personalize learning, provide engaging content, streamline assessments, and make resources more accessible”. RN15 views, “AI offers opportunities to make learning more personalized, efficient, and aligned with the needs of both students and educators”. Furthermore, RN12 confirms, “AI offers personalized learning opportunities”. To finish up, RN14 claims, “AI has the potential to personalize learning to individual learner needs”, while “RN16, views, “AI powered learning tools can make language learning more interactive and individualized at the same time”. RN21 reports, “AI-powered chatbots provide personalized learning experiences, making language practice more interactive and effective. I tried to incorporate some of these tools in my teaching and they were very helpful”.

AI also provides a rich environment for EFL learners to interactively practice of language. RN7 points out, “AI provide interactive language practice through a number of tools and apps, and automated writing evaluation tools”. Furthermore, RN2 adds, “Students for example interact with AI in conversation practice, getting replies to their queries. Even they can get simple evaluation to their productions”. This idea is also supported by RN 17 who mentions, “AI provides effective classroom interaction that aims to achieve the desired learning outcomes”. EFL instructors can also interact with their students using AI. This is confirmed by RN10 who proposes, “It will also help teachers with grading, attendance, and tracking, allowing them to focus more on teaching some topics and interacting with students”.

AI tools participate in ELT practices especially for the four language skills and language systems. RN 19 points out, “NLP have significantly enhanced language skills in reading, writing, listening, and speaking. In writing, AI helps correct grammar, improve style, and even generate content, making it a valuable tool for writers, students, and professionals. For listening, AI-based speech recognition systems, such as those used in transcription services and virtual assistants, have improved the accuracy and real-time understanding of spoken language. Speaking skills are supported by AI through language learning apps, conversational agents, and real-time translation tools that help users practice pronunciation and engage in multilingual communication”. RN 20 opines, “twee.com and diffit.me. platforms are useful for reading, writing and listening skills. They also suggest some speaking activities (e.g., questions that students can ask each other). Alternatively, to improve speaking skills, Copilot now features the possibility to talk directly to the chatbot and receive feedback in audio form. The same goes for PI AI”. RN22 mentions, “I use AI in teaching writing and speaking. For writing, AI helps with grammar and spelling corrections. For speaking, AI can generate role-play scenarios, provide pronunciation feedback, and facilitate interactive conversations to improve fluency”. To sum up, RN17 reports, “AI offers interactive learning tools”.

Adopting AI also makes EFL learners to learn and practice English wherever they are; this ensures their productivity. RN15 shows, “New, engaging and interactive content can be created using AI parsed learner needs and interests”. RN14 adds, “Practice opportunities can now go beyond the limited reach of the classroom with the use of AI tools and apps”. Furthermore, RN11 utters, “AI enhances the learning experience, improve learning outcomes, and increase the productivity and efficacy of instructors”. Furthermore, RN1 opines, “AI has affordances to support in English language teaching and learning ELT/L”. Finally, RN6 views, “AI improves students’ skills and abilities and can be used not only in teaching language process but also in assessing students’ progress/achievements”.

AI is a powerful tool for improving language skills. It helps with speaking through pronunciation feedback (e.g., Elsa Speak) and writing by correcting grammar and style as in QuillBot. For reading, AI-assisted apps like LingQ and Readlang enhance comprehension. Listening skills improve with adaptive exercises from platforms like BBC Learning. Additionally,

AI is valuable in automatizing feedback and assessment. RN12 affirms, “AI offers automated assessment and feedback. This feedback can be utilized by EFL instructors. For instance, RN10 reports, “[EFL]teachers will heavily rely on AI to assist with assessment, feedback, and other tasks”. Similarly, RN17 asserts, “teachers will heavily rely on AI to give feedback”. This automaticity is something never applied before. RN14 adds, “Using automation, language assessment can be streamlined like never before”. RN1 links the students’ needs with AI supports stating, “the adaptive nature of AI tools was valued for its ability to cater to individual needs and offer immediate feedback”. RN 18 adds “The rapid evolution of AI tools like ChatGPT, Copilot, Claude, Gemini, DeepSeek, and Grok is indeed shaping new morphosyntactic patterns in language use. These tools are not only enhancing language learning but also influencing how we communicate and structure our sentences”. To finish up, RN7 points out, “These [supports] include personalized learning experiences suitable to individual student needs, instant feedback on writing exercises”.

AI plays a major role in developing and preparing materials. RN17 mentions, “AI enhances materials creation”. This idea is confirmed, “teachers will heavily rely on AI in material creation, “RN10 reported. RN6 adds, “AI can be considered as one of the vital supplementary teaching materials”. EFL learners can use AI to produce content. RN12 views, “AI will enable learners to construct knowledge by creating multimodal content”. Preparing content using AI may be engaging and motivational. RN8 suggests, “AI technology offers engaging content and makes resources more accessible”. RN 20 argues, “twee.com and diffit.me. Both platforms allow for the creation of teaching materials by inputting a topic or by pasting a YouTube-video or the link of a web article”.

These important opportunities are shaping the future of AI-powered learning. Our findings aligned with the many research findings. For instance, Hang et al. [21] confirmed the indispensable role of AI-powered paradigms in EFL teaching and learning. Our findings agree with Winaitham [27] who stated that AI applications provide adoptive learning, automatic feedback, and provide chance for language practicing. Furthermore, Ulfa [1] pointed out that AI provides tutoring systems which engage learners in game-based learning settings. Furthermore, Amin [16] affirmed that AI technology provides potential for personalized learning where students can find themselves and learn at their own pace. The study also scrutinized the roles of EFL instructors in AI-powered environment. This will be presented herein.

## Conclusion

This paper investigated the potential opportunities of AI in the context of EFL higher education from the perspective of language instructors where data was collected through a focused group discussion on ResearchGate. Using thematic analysis, the analysis showed that AI technologies offer significant benefits, such as personalized learning and immediate feedback, instructors remain crucial in providing emotional support and fostering critical thinking. The role of English language instructors is evolving with the integration of AI in education. By understanding instructors’ perspectives and addressing the associated challenges, educational institutions can enhance the collaboration between AI tools and language instructors, ultimately improving learning outcomes. Findings of this study are crucial for EFL instructors and students. Instructors are required to develop themselves and their teaching abilities in a way parrelling with the great advance in AI technologies. Institutions were also supposed to update teachers’ knowledge and skills to make use of the AI technologies without being awkward.

This paper acknowledges several limitations. First, the research relied on focused group discussions, which may not fully capture the diversity of instructors’ experiences and perceptions across broader contexts. Second, the study primarily focused on the opportunities associated with AI integration in ELT, leaving other critical areas, such as students’ perspectives and institutional readiness for AI adoption, unexplored. Finally, the evolving nature of AI technology means that findings may need continual updates to reflect technological advancements.

Future research could address these limitations investigating how students perceive and interact with AI-powered tools in ELT, including their impact on learning outcomes and motivation. Other studies could analyse the policies, resources, and training required for successful AI integration in educational settings. By addressing these areas, future research can provide a more comprehensive understanding of AI’s potential and transformational roles in ELT, guiding effective and sustainable integration in educational systems.

## Supporting information

S1 Appendix ResearchGate details (uploaded separately). (DOCX)

S2 Appendix Summary of the demographic and professional details of the participants (uploaded separately). (DOCX)

S1 File Supporting Information-2. (DOCX)
