# Supplementary material for: AI powered ELT: Instructors’ transformative roles and opportunities
Source: PLoS One. 2025 May 29;20(5):e0324910. doi: 10.1371/journal.pone.0324910 (PMC12121750; doi:10.1371/journal.pone.0324910)
Supplement: S1 Appendix — (DOCX) [file pone.0324910.s001.docx]

**Appendix 1**

ResearchGate detail**s**

| RQ | Questions | Posting date | Reads | Followers | Replies | Recommendations |
| --- | --- | --- | --- | --- | --- | --- |
| RQ1 | AI and Roles of Language instructors | 14/8/2024 | 365 | 5 | 5 | 3 |
| RQ2 | AI and the future of English language teaching | 2/7/2024 | 697 | 22 | 29 | 7 |
|  | Practices | 9/3/2025 | 28 | 5 | 4 | 2 |
