# Supplementary material for: AI powered ELT: Instructors’ transformative roles and opportunities
Source: PLoS One. 2025 May 29;20(5):e0324910. doi: 10.1371/journal.pone.0324910 (PMC12121750; doi:10.1371/journal.pone.0324910)
Supplement: S2 Appendix — (DOCX) [file pone.0324910.s002.docx]

**Appendix 2**

Summary of the demographic and professional details of the participants

| Code | University | Degree | Country |
| --- | --- | --- | --- |
| RN1 | Technical College Požarevac | Full professor -retired | Serbia |
| RN2 | Hadhramout University | Associate Professor of Applied Linguistics | Yemen |
| RN2 | University of Diyala | Professor | Iraq |
| RN4 | Albaydha University | Assistant Lecturer | Yemen |
| RN5 | Manisa Celal Bayar University | Doctor of Philosophy | Turkey |
| RN6 | University of Aden | Assistant prof. of applied linguistics | Yemen |
| RN7 | Arab Open University | PhD Assistant  Professor | Saudi Arabia |
| RN8 | Lecturer | Universitas Lancang Kuning Pekanbaru | Indonesia |
| RN9 | A'Sharqiyah University | Doctor of Arts | Oman |
| RN10 | University of Technology and Applied Sciences | PhD in Applied Linguistics | Oman |
| RN11 | University of Tripoli | Assistant Professor | Libya |
| RN12 | UiB | Associate Prof. of Applied Linguistics | Norway |
| RN13 | Ibb University | Professor | Yemen |
| RN14 | Qassim University | Professor | Saudi Arabia |
| RN15 | Queen Arwa University | Assistant Professor of Linguistic | Yemen |
| RN16 | Sushila Devi Bansal College of Technology | Professor | India |
| RN17 | Universiti Tenaga Nasional (UNITEN) | PhD | Iraq |
| RN18 | University of Bergen | Associate Professor | Norway |
| RN 19 | Iran University of Science and Technology | Master of Technology Management | Iran |
| RN 20 | University of Camerino | Professor (Full) | Italy |
| RN 21 | King Khalid | PhD Candidate | Saudi Arabia |
| RN 22 | University of New England | PhD Candidate | Australia |
