# Supplementary material for: AI powered ELT: Instructors’ transformative roles and opportunities
Source: PLoS One. 2025 May 29;20(5):e0324910. doi: 10.1371/journal.pone.0324910 (PMC12121750; doi:10.1371/journal.pone.0324910)
Supplement: S1 File — (DOCX) [file pone.0324910.s003.docx]

**Data Collection on ResearchGate**

**AI and the future of English language teaching | ResearchGate**

**AI and the future of English language teaching**

Discussion

- Started July 2, 2024

Dear Colleagues,

As AI technology continues to evolve, the role of language instructors is undergoing significant changes.

In your opinion, what future opportunities do you foresee for AI in enhancing English language teaching at universities?

Your insights and contributions to this discussion are greatly valued.

Reply to this discussion

Teaching English as a Foreign Language

Artificial Intelligence

Following

Share

Popular replies (2)

**XXXXXXXXXX** added a reply

July 4, 2024

**Artificial Intelligence (AI) Solutions In English Language Teaching: Teachers-Students Perceptions And Experiences**

"This literature research article explores the perceptions and experiences of teachers and students regarding Artificial Intelligence (AI) solutions in English language teaching. With the rapid advancements in AI technologies, there is a growing interest in leveraging these tools to enhance language learning experiences. Understanding the perspectives of teachers and students is crucial for successful implementation and to harness the potential benefits of AI in the field of English language education. The study adopts a mixed-methods research approach, incorporating both quantitative and qualitative methods. A survey was conducted to gather quantitative data on participants' attitudes, beliefs, and experiences related to AI integration. Additionally, in-depth interviews and focus group discussions were conducted to obtain qualitative insights and delve into participants' perceptions and challenges. The findings of the study reveal positive attitudes towards AI solutions in English language teaching, with participants highlighting the effectiveness of AI technologies in improving language skills and providing personalized instruction. The adaptive nature of AI tools was valued for its ability to cater to individual needs and offer immediate feedback. However, concerns were raised regarding technological readiness and the need for training and support in effectively utilizing AI solutions. These findings have implications for educators, policymakers, and curriculum developers, highlighting the need for technological readiness, teacher training, and support in implementing AI solutions effectively. By embracing the potential of AI while preserving the human element, English language teaching can benefit from personalized and adaptive learning experiences..."

Recommended

Share

- 8 Recommendations

**XXXXXXXXXX**  added a reply

July 2, 2024

I think that AI could occupy some roles of EFL teachers. Students for example interact with AI in conversarion practice, getting replys to their queries. Even they can get simple evaluation to their productions. I think the teachers role will be minimized to evaluation students essays, research papers and prrsentation.

… Read more

Recommended

Share

- 6 Recommendations

All replies (29)

**XXXXXXXXXX**  added a reply

August 12, 2024

**Navigating the Transformative Impact of Artificial Intelligence on English Language Teaching: Exploring Challenges and Opportunities**

"This study aims to examine the impact of artificial intelligence (AI) on English language teaching in higher education. The scope of the study was limited to the opportunities and challenges of English language teaching...

AI has changed the landscape of English language teaching in higher education. English lecturers utilized different types of AI for various purposes, for example, asking and solving questions and checking for grammatical errors, checking plagiarism, paraphrasing, and reviewing literature. The study also revealed that AI has a variety of advantages for language teaching and learning, including the detection of plagiarism and grammatical errors. In addition, AI has created opportunities and challenges for the future of English language teaching. AI required digital literacy to utilize. The English teaching profession might be taken over by AI in the future, so English lecturers must continuously improve their digital literacy..."

Recommend

Share

- 4 Recommendations

**XXXXXXXXXX**  added a reply

August 12, 2024

I believe that AI has the potential to make English language teaching more efficient, personalized, and engaging, ultimately leading to better outcomes for students. With AI-powered platforms, students can receive immediate feedback on their work, enabling them to quickly correct errors and concentrate on areas requiring improvement. on

… Read more

Recommended

Share

- 2 Recommendations

**XXXXXXXXXX**  added a reply

August 17, 2024

AI will significantly enhance English language teaching at universities as learners will easily get access to real-life interactions so they can practice listening and speaking when they are out of the class.

AI can help instructors in creating the material. It can also help in assessing the performance of the trainees.

By providing for example speech- to- text services,AI will support the disabled learners. .

Share

- 2 Recommendations

**XXXXXXXXXX**  added a reply

August 17, 2024

Another research article is available about this issue.

**Generative AI and AI Tools in English Language Teaching and Learning: An Exploratory Research**

"Generative AI (GenAI) tools such as ChatGPT, Gemini, and Copilot have created concerns in academia, particularly after the launch of ChatGPT. GenAI and AI have been the buzz words and academics are discussing about the possibilities of its positive and negative impacts on educations and research. Recently, studies have been conducted on the influence of GenAI tools in education and research. With the above concerns and the impact of GenAI, grounded on Vygotsky's Zone of Proximal Development (ZPD) as a theoretical lens, this study explores how English language teachers integrate GenAI tools to enhance teaching and learning. Particularly, this study explores the integration of GenAI tools in English language teaching and learning, focusing on teaching efficiency, student engagement, personalized learning, and writing skills, subscribing to exploratory research methods grounded on semi-structured interviews. The findings of the study affirmed the positive impact of GenAI tools on teaching efficiency, students’ engagement, and writing skills. The results indicated that GenAI positively influences teaching efficiency and student engagement in learning. The implications of this research highlighted the potential of GenAI tools to create a more intelligent and personalized learning environment for English language teaching that benefits both educators and learners..."

Article Generative AI and AI Tools in English Language Teaching and ...

Recommended

Share

- 4 Recommendations

**XXXXXXXXXX**  added a reply

August 26, 2024

**Artificial Intelligence in Teaching English Language in Libya**

**XXXXXXXXXX**

**General Background**

Since its rising before 3 years, a large number of stories about AI as a great technology that may change the world. There were many talks about its superiority to Google and other similar technologies. There was big fear about the future of humanity and labor force, and what people can do when they get fired from their jobs because AI can do all jobs in all fields.

One of the fields that AI can play an important role in is the field of education. Teachers at their schools or universities feared a lot to be fired and replaced with AI robots. they expect that AI can compensate for them and even the cost of teachers will decrease which makes governments take a lot of care with AI and research in this field.

Currently, it seems that the world gets rid of its fear from this advanced technology. Some universities in the world have launched departments for AI. In some Arabic countries, people are still talking about fears from the future with AI.

**AI in Teaching English**

Anyhow, there some good trends in the field of AI. It has a lot of benefits in many fields. Here, we are limited to the field of teaching English language. Students of English language can upgrade themselves and excel their levels in different fields. They can meet their individual needs.

AI can help grading task and other works. It also makes a lot of free time for teachers, so that they can perform other tasks and they can focus of important affairs for their students such as planning their lessons and developing the thinking of their students.

It also can help in the lessons of grammar, vocabulary and writing and of course their conversation. It can enhance the performance of the students. Students and their teachers can benefit from their colleagues in different parts of the world, regardless of the language.

As Libyan public schools suffer a lot of problems specially in teaching English language, which negatively affect the performance of the students and their acquisition of English language, they can benefit from AI in different ways. They can benefit from virtual tutors and lessons. This is called Interactive Learning.

In the field of graduate studies, AI can hep students by suggesting topics for their research studies which lead to obtain their MA or PhD degrees. In the field of research, AI can analyze large datasets which may help and share in conducting researches. It can also generate drafts of research papers.

Recommend

Share

- 3 Recommendations

**XXXXXXXXXX**  added a reply

September 6, 2024

GPTAvatar has been extended to an **authoring tool**. It enables creating your **own avatar configurations** online in the browser in many languages and with any content you want. This extended version also creates **log data for research**.

YouTube Video:

Link:

(Run it in Chrome or Edge, not in Firefox, give Microphone permissions and wait 1-2 Minutes)

Website and documentation:

Recommend

Share

- 1 Recommendation

**XXXXXXXXXX**  added a reply

September 17, 2024

**Study provides evidence of AI’s alarming dialect prejudice**

"While large language models (LLMs) like ChatGPT-4 have been trained to avoid answers that overtly racially stereotype, a new study shows that they “covertly” stereotype African Americans who speak in the dialect prevalent in New York, Detroit, Washington DC and other cities such as Los Angeles. In “AI generates covertly racial decisions about people based on their dialect” published in *Nature* at the end of August, a team of three researchers working with Dr Valentin Hofmann at the Allen Institute for AI in Seattle shows how AI’s (learned) prejudice against African-American English (AAE) can have harmful and dangerous consequences...

The study, the authors write, “provides the first empirical evidence for the existence of dialect prejudice in language models: that is, covert racism that is activated by features of a dialect (AAE).” The study states: “Using our new method of matching guise probing, we show that language models exhibit archaic stereotypes about speakers of AAE that most closely agree with the most negative human stereotypes about African Americans ever experimentally recorded, dating from before the civil rights movement.”..."

Recommend

Share

- 2 Recommendations

**XXXXXXXXXX**  added a reply

October 14, 2024

**AI for Language Education**

"The Artificial Intelligence for Language Education (AI Lang) project is an ECML-funded initiative that aims to document how teachers and learners use AI in language education, what competences teachers (need to) draw on, and how language teachers and learners can use AI efficiently, appropriately and ethically.

At this stage of the project, we are acquainting ourselves with the various AI-assisted language education tools that teachers use in their classes. We have already completed an online survey from which we gained some insight about the pedagogical applications of AI, the concerns teachers report, and their needs.

What we want to do next is deepen this knowledge by learning more from specific examples of AI-assisted language education and by exchanging ideas with people who have used AI creatively in their classes..."

Recommend

Share

- 3 Recommendations

**XXXXXXXXXX**  added a reply

February 11

AI is going to be the future of English teaching, with learning tailored to students' needs, automation of assessments, and increased student engagement. AI-driven chatbots, speech recognition, and adaptive learning platforms go an extra mile in providing immediate feedback that helps in correcting pronunciation, grammar, and vocabulary. Virtual tutors and AI-powered language models make learning even more interactive and accessible, keeping in view different learning paces. In addition, AI can track students' progress and suggest customized lessons, making learning more effective. As AI progresses, it will complement the role of teachers through automation of routine administrative tasks, creating immersive language learning experiences fueled by data.

**XXXXXXXXXX**

Recommend

Share

- 2 Recommendations

**AI and Roles of Language instructors**

Discussion

- Started August 14, 2024

Dear language instructors,

AI has been used in all aspects of life including education:

How do you forsee the future roles of EFL instructors in AI powered higher education?

… Read more

Reply to this discussion

Artificial Intelligence

Following

Share

All replies (5)

**XXXXXXXXXX**  added a reply

August 14, 2024

EFL instructors are responsible for designing materials, creating content, building life skills, and explaining the ethical and unethical use of such AI applications to their students.

… Read more

Recommend

Share

**XXXXXXXXXX**  added a reply

August 14, 2024

Language instructors play a critical role in fostering communication, cultural understanding, and nuanced language skills that AI alone cannot fully replicate. They guide students through complex linguistic concepts, idiomatic expressions, and contextual use of language, ensuring learners develop not only technical proficiency but also cultural competence. Instructors also offer emotional support, motivation, and encouragement, which are crucial for language learning—a process often fraught with frustration and setbacks. AI can assist instructors by automating administrative tasks like grading and assessment, freeing up time for personalized teaching. AI-driven platforms can analyze student progress, offering insights into specific areas where students may struggle, allowing instructors to tailor lessons to meet individual needs. Moreover, the role of instructors is evolving rather than diminishing. They are becoming facilitators who use AI to enhance the learning experience, designing interactive, immersive, and student-centered activities that AI cannot replicate. By leveraging AI for routine tasks, instructors can focus on fostering critical thinking, creativity, and deep engagement in the language, maintaining a balance between technological efficiency and human connection in education.

Recommend

Share

**XXXXXXXXXX**  added a reply

August 15, 2024

The role of the teacher could shift from delivering traditional lessons to being a facilitator who helps students explore content and interact with AI systems. The teacher will be the one who encourages discussions and promotes critical thinking, and AI tools can be used to analyze student progress and identify their strengths and weaknesses, allowing them to guide each student on an individual learning path that suits their needs. **XXXXXXXXXX**

Recommend

Share

- 1 Recommendation

**XXXXXXXXXX**  added a reply

August 16, 2024

AI will not replace the teachers but help them modify and adjust their teaching methods, techniques, and strategies. In a word, though AI handles many routine tasks, the human touch of EFL instructors will remain indispensable in fostering supportive and effective learning.

… Read more

Recommend

Share

**XXXXXXXXXX**  added a reply

September 12, 2024

AI will enhance the role of teachers, making teaching easier and more efficient. They will serve as facilitators and supervisors, guiding students to learn correctly and supervising those who are proficient learners and users of AI. Additionally, they will act as monitors, ensuring adherence to ethical concerns and preventing any violations.

… Read more

Recommend

Share

- 1 Recommendation

**AI and the current practices of English language teaching | ResearchGate**

**AI and the current practices of English language teaching**

Discussion

- Started March 9

Dear Language Instructors,

Almost nine months ago, I posted a question about AI and the potential of English language teaching. Now, can we update our discussion about AI and the current practices with specific attention to language skills: (Reading, writing, listening and Speaking)?

Yes, nine months is a short period, but with the advances of AI technology, I am sure that you have experienced many opportunities with AI tools in language skills.

Your discussion is highly appropriated.

Abduljalil

Reply to this discussion

Artificial Intelligence

Teaching English as a Foreign Language

Periodicals

Following

Share

All replies (4)

**XXXXXXXXXX**  added a reply

March 9

Yes, AI continues to transform language education by providing personalized, adaptive, and accessible learning experiences. The rapid evolution of AI tools like ChatGPT, Copilot, Claude, Gemini, DeepSeek, and Grok is indeed shaping new morphosyntactic patterns in language use. These tools are not only enhancing language learning but also influencing how we communicate and structure our sentences.

Recommend

Share

**XXXXXXXXXX**  added a reply

March 9

Absolutely! As someone who works in the field of AI (not a language instructor), current approaches in AI, especially with advances in natural language processing (NLP), have significantly enhanced language skills in reading, writing, listening, and speaking. AI-based tools like GPT-4 and other large language models excel at understanding, allowing for efficient summarization and extraction of information from large amounts of text. In writing, AI helps correct grammar, improve style, and even generate content, making it a valuable tool for writers, students, and professionals. For listening, AI-based speech recognition systems, such as those used in transcription services and virtual assistants, have improved the accuracy and real-time understanding of spoken language. Speaking skills are supported by AI through language learning apps, conversational agents, and real-time translation tools that help users practice pronunciation and engage in multilingual communication. These advances make language-related tasks more accessible, efficient, and personalized, although challenges such as bias, context understanding, and ethical concerns remain areas of ongoing research and development.

I would appreciate it if you would read my articles and recommend them to increase their visibility.

Recommend

Share

**XXXXXXXXXX**  added a reply

March 11

Hello. Apart from chatbots, the following AI-driven platforms are used intensively by lecturers (especially in secondary schools):

twee.com and diffit.me. Both platforms allow for the creation of teaching materials by inputting a topic or by pasting a youtube-video or the link of a web article. These platforms are useful for the reading, writing and listening skills. They also suggest some speaking activities (e.g., questions that students can ask each other). Alternatively, to improve speaking skills, Copilot now features the possibility to talk directly to the chatbot and receive feedback in audio form. The same goes for PI AI. The difference between the two is that Copilot can be used via a PC (and users must have a microsoft account to log on), whereas PI AI has developed an App which is to be downloaded to users' smartphones. In this way, users can have conversation (in English) with the chatbot and, perhaps, ask to be corrected when they make "mistakes".

Recommended

Share

- 1 Recommendation

**XXXXXXXXXX**  added a reply

March 11

Thank you for your contribution to this discussion

… Read more

Share
